# VEGF Gene Expression in Adult Human Thymus Fat: A Correlative Study with Hypoxic Induced Factor and Cyclooxigenase-2

**DOI:** 10.1371/journal.pone.0008213

**Published:** 2009-12-14

**Authors:** Francisco Tinahones, Julian Salas, María Dolores Mayas, Adrian Ruiz-Villalba, Manuel Macias-Gonzalez, Lourdes Garrido-Sanchez, Manuel DeMora, Inmaculada Moreno-Santos, Rosa Bernal, Fernando Cardona, Rajaa El Bekay

**Affiliations:** 1 Centro de Investigación Biomédica en Red, Fisiopatología de la Obesidad y Nutrición (CB03/06), Instituto Carlos III, Madrid, Spain; 2 Servicio de Endocrinología, Hospital Virgen de la Victoria, Málaga, Spain; 3 Departamento de Cirugía Cardiovascular, Hospital Carlos Haya, Malaga, Spain; 4 Fundacion Instituto Mediterráneo para el Avance de la Biotecnología y la Investigación Sanitaria, Laboratorio de Investigación Biomédica, Hospital Virgen de la Victoria, Malaga, Spain; 5 Departamento de Enfermedades Cardiovasculares, Hospital Carlos Haya, Málaga, Spain; New Mexico State University, United States of America

## Abstract

It is well known that the adult human thymus degenerates into fat tissue; however, it has never been considered as a potential source of angiogenic factors. Recently, we have described that this fat (TAT) produces angiogenic factors and induces human endothelial cell proliferation and migration, indicating its potential angiogenic properties.

**Design:**

Adult thymus fat and subcutaneous adipose tissue specimens were obtained from 28 patients undergoing cardiac surgery, making this tissue readily available as a prime source of adipose tissue. We focused our investigation on determining VEGF gene expression and characterizing the different genes, mediators of inflammation and adipogenesis, and which are known to play a relevant role in angiogenesis regulation.

**Results:**

We found that VEGF-A was the isoform most expressed in TAT. This expression was accompanied by an upregulation of HIF-1α, COX-2 and HO-1 proteins, and by increased HIF-1 DNA binding activity, compared to SAT. Furthermore, we observed that TAT contains a high percentage of mature adipocytes, 0.25% of macrophage cells, 15% of endothelial cells and a very low percentage of thymocyte cells, suggesting the cellular variability of TAT, which could explain the differences in gene expression observed in TAT. Subsequently, we showed that the expression of genes known as adipogenic mediators, including PPARγ1/γ2, FABP-4 and adiponectin was similar in both TAT and SAT. Moreover the expression of these latter genes presented a significantly positive correlation with VEGF, suggesting the potential association between VEGF and the generation of adipose tissue in adult thymus.

**Conclusion:**

Here we suggest that this fat has a potential angiogenic function related to ongoing adipogenesis, which substitutes immune functions within the adult thymus. The expression of VEGF seems to be associated with COX-2, HO-1 and adipogenesis related genes, suggesting the importance that this new fat has acquired in research in relation to adipogenesis and angiogenesis.

## Introduction

It has been shown that the thymus grows rapidly during embryonic life and during childhood, reaching its maximum absolute size in puberty; thereafter growth ceases and involutes gradually until old age, when the gland is often smaller than at birth [1]. This age involution is shown by a decrease in the overall weight of the organ, associated lymphoid tissue atrophy and replacement by mature adipose tissue [1]. For this reason, all the studies carried out on adult thymus involution have been focused primarily on the immunological aspect, but has never been considered interesting enough to be studied as a potential source of humoral and angiogenic factors. Part of this tissue is discarded in aortic cannulation procedures of the ascending aorta in cardiovascular surgery in patients in need of a cardiopulmonary bypass (CPB), making this tissue a readily available prime source of adipose tissue.

Vascular endothelial growth factor (VEGF) is the first member of angiogenic factors to be cloned and remains today the best-characterized angiogenic growth factor [2]. VEGF was first described as a vascular permeability factor [3] but has also been recognized as being a potent stimulator of endothelial proliferation and migration [4]. Gene targeting studies have demonstrated the critical importance of VEGF for both vasculogenesis and angiogenesis [5]. It has been demonstrated that white adipose tissue produces and secretes many different types of proangiogenic factors, such as VEGF-A and VEGF-B, the two key angiogenic factors produced by adipocytes [6]. Other adipose-tissue derived factors with pro-angiogenic properties are VEGF-C and VEGF-D, which have been found to be important in the proper formation and maintenance of the lymphatic network [7]. As a result, VEGF has been used in numerous preclinical and early clinical gene or protein therapy trials with varying degrees of success [8].

We have recently found that thymus fat produces a variety of angiogenic factors such as Angiopoietin (Ang), Tie2, VEGF, VEGF-R1 and VEGF-R2. Furthermore, human umbilical cord endothelial cells cultured in the presence of thymus fat extract displayed enhanced proliferative and migratory responses, which are two relevant steps in angiogenesis [9]. These findings lead us to believe that TAT is an interesting source of angiogenic factors which may, in the future, have a relevant role in promoting angiogenesis and tissue repair. To complete our previous study [9], we performed a comparative evaluation of VEGF expression in human thymus (TAT) and subcutaneous (SAT) adipose tissues. As angiogenic regulation is usually associated with inflammatory and anti-inflammatory mechanisms involving cyclooxygenase-2, heme oxygenase-1 and hypoxia-induced factor-1 transcription factors, we tested the hypothesis that VEGF expression could correlate with these markers.

## Materials and Methods

### Patients and Adipose Tissue Collection

This study was approved by the local ethical committee of Hospital Carlos Haya, and signed informed consent was obtained from all participating patients by the Spanish Society of Thoracic and Cardiovascular Surgery (SECTCV). The number of patients studied was 28. The mean age was 70.2±2.7 years. All patients received a coronary-artery bypass graft (CABG) with cardiopulmonary bypass (CPB). The mean number of grafts used was 3.1 per patient. The relevant clinical and metabolic characteristics of these subjects are shown in supplemental Table S1. Both subcutaneous and thymus adipose tissues were obtained at the beginning of the procedure. The site of subcutaneous fat harvest was from the chest incision. Parts of these tissues were stored at −80°C, and the remainder was processed for the immunohistochemistry study.

### Real-Time PCR

The amplifications were performed using a MicroAmp optical 96-well reaction plate (PE Applied Biosystems) on an ABI 7500 Real-Time PCR System (Applied Biosystems). RT qPCR reactions were carried out for all genes using specific *TaqMan® Gene Expression Assays.* During PCR the Ct values for each amplified product were determined using a threshold value of 0.1. The specific signals were normalized by constitutively expressed cyclophilin (Cyc) signals using the formula 2-ΔCt.

### Preparation of Nuclear and Cytopasmic Protein Extracts

The thymus tissue sample (100 mg) was homogenized in cold phosphate-buffered saline (PBS), containing protease inhibitors, and the homogenate was centrifuged at 12,000×g for 45 min to obtain a clear crude extract, and the resulting pellet was lysed with NE-PER *Nuclear and Cytoplasmatic Extraction Reagents protocol* (Pierce Biotechnology). Briefly, 200 µl of buffer CER I supplemented with protease inhibitors were added to the homogenate and then sonicated on ice for 20 seconds and then incubated on ice for a further 10 min. The homogenate with 10 µl of CER II buffer was centrifuged at 12,000×g for 10 min at 4°C. The supernatant, which represents a cytoplasmic fraction, was separated from the pellet, which represents nucleic fraction. This was then resuspended and sonicated on ice in 50 µl of NER buffer and then centrifuged for 10 min at 12,000×g at 4°C. The supernatant represents nucleic soluble fraction. Both fractions of the cytosolic and nuclear extracts were stored at −80°C.

### Western Blot Analysis

Protein extracts (30 µg) were separated by SDS-PAGE, blotted onto a PVDF membrane and then incubated with specific antibodies. Protein signals were detected by electrochemiluminescence detection Quantity One ® software (Bio-Rad Laboratories).

### Electrophoretic Mobility Shift Assay (EMSA)

Oligonucleotides were 3′ end-labeled with Biotin using terminal deoxynucleotidyl transferase (TdT), according to the *Biotin 3′ End DNA Labeling Kit* protocol (Pierce). EMSA was performed using double-stranded oligonucleotide probes spanning positions +563 to +575 of the VEGF promoter region containing both HRE (binding site for hypoxia inducible factor-1, [HIF-1]) (+533 to +549). Labeled oligonucleotides were incubated with nuclear lysate from different AT's (10 µg total protein) for 20 min at room temperature and separated by electrophoresis on a 6% nondenaturing polyacrylamide gel. DNA-protein complexes were transblotted onto nylon membranes, developed by ECL using the *LightShift® Chemiluminescent EMSA Kit* (Pierce). For super shift, proteins were incubated with anti-HIF-1α and anti-p65 mAb for 1h at 4°C prior to the binding assay.

### Immunohistochemistry of Adipose Tissue

For the immunostaining process, adipose tissue sections (25µm) were first fixed for 30 minutes with formol, and washed in 0.1 M PBS, pH 7.4 for 15 minutes. The sections were treated with PBS containing 10% methanol and 10% hydrogen peroxide for 30 min to quench endogenous peroxidase activity and then exposed overnight to the primary antibodies. Washed sections were then incubated with the appropriate biotinylated secondary antibodies, immunoglobulin (Ig) was applied for 1 h and 30 min for Extravidin-peroxidase. The color was developed by adding peroxidase substrate 3,3′-diaminobenzidine. Sections were counterstained with Mayer's Hematoxylin and, finally, mounting solution and cover slips were added.

### Flow Cytometry

Cells were labeled with monoclonal antibodies to cell surface markers directly fluorochrome-conjugated and fixed in 1% paraformaldehyde. All the antibodies were fluorescein isothiocyanate (FITC)-conjugated (CD11b, ITGAM, EMR1, CD45, CD31 CD49, CD8, CD4 and FABP-4). Samples were acquired immediately or stored at 4°C and acquired within 36 h. The labeled cells were washed and re-suspended in PBS. Percentage of positive cells, which indicates the ratio of positive-to- background staining, was analyzed on a dual-laser BD FACSCalibur™.

### Statistical Analysis

SPSS Inc. software (Version 15.0) was used for all statistical analyses. Comparisons between the normalized mRNA levels of different tissues were made by means of ANOVAs test. The correlation analysis of mRNA quantitative expression by each of the genes was performed with Pearson's correlation coefficient Test (*r*).

## Results

### VEGF Isoform Levels in Human Thymus and Subcutaneous Adipose Tissues

**Figure 1:**
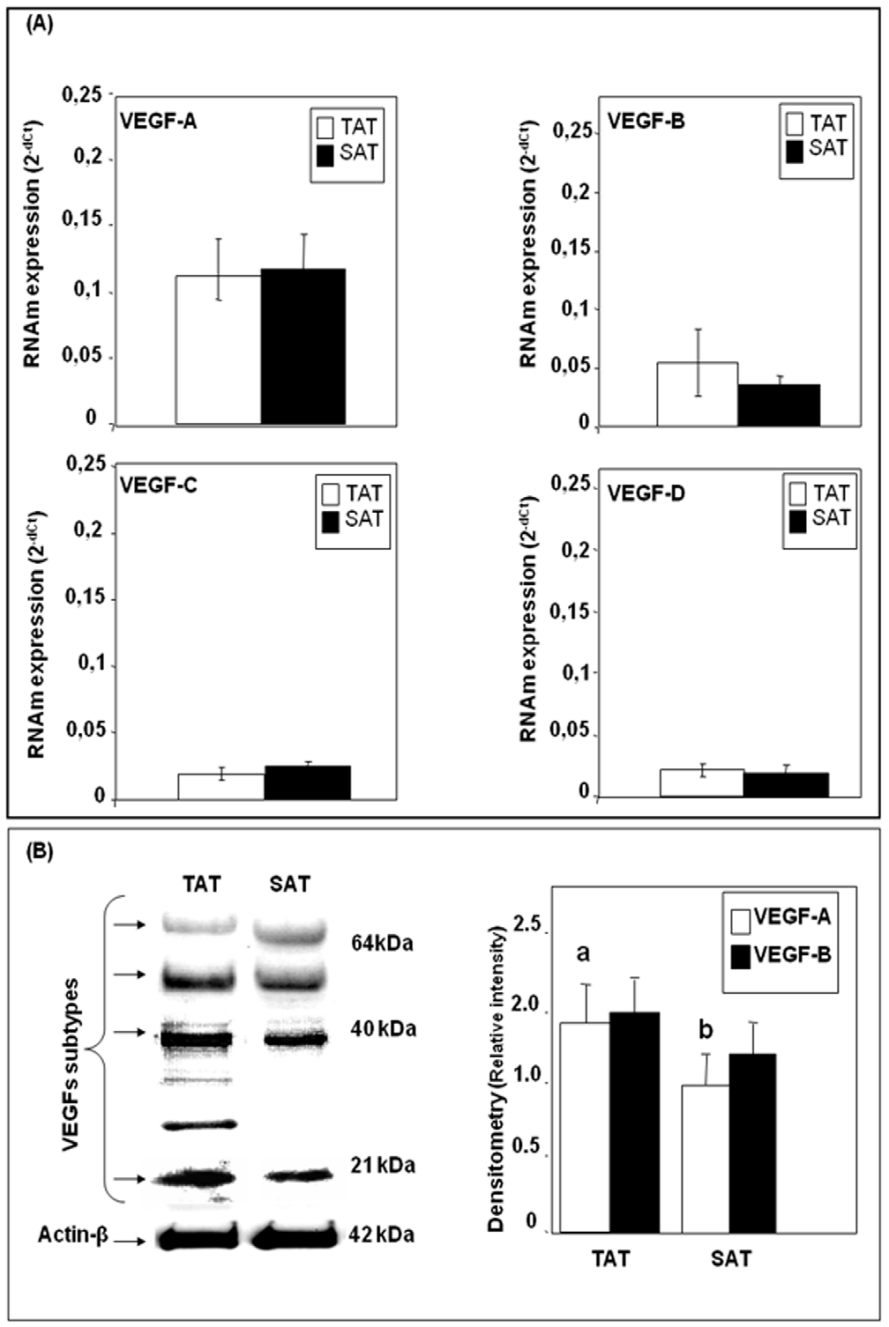
VEGF isoforms mRNA and protein expression in TAT and SAT. TaqMan® real time PCR for VEGF isoforms (VEGF A, VEGF-B, VEGF-C and VEGF-D) was performed on human thymus (TAT) and subcutaneous (SAT) adipose tissues. VEGF isoforms mRNAs were normalized to cyclophilin (Cyc) levels (A). Results were expressed as the mean±SEM of the completed experiment in triplicate (n = 26). Immunoblot analysis of VEGF isoforms. Densitometry analyses are presented as a relative ratio of VEGF isoforms to actin-β. Bars represent mean±SEM from 6 samples per adipose tissue type. Bars with different letters have a significant difference, P<0.05 (B).

**Figure 2:**
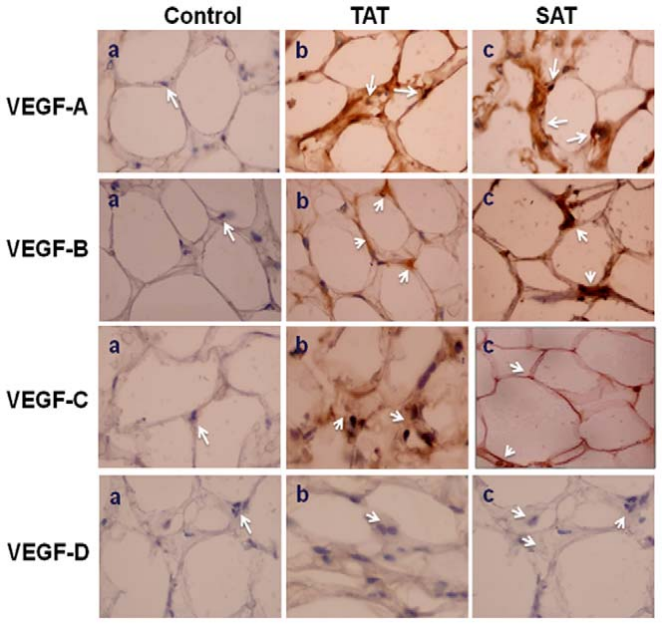
Immunohistochemical identification of VEGF isoforms. (a) Negative controls with blue labeling corresponding to haematoxylin labeled nucleus (**arrows**). (b) VEGF-A, VEGF-B, VEGF-C and VEGF-D positive cells from human thymus adipose tissue **(arrows)**. (c) VEGF-A, VEGF-B, VEGF-C and VEGF-D positive cells from human subcutaneous adipose tissue (**arrows**). Positive cells shown with brown labeling.

qPCR analysis showed that VEGF-A was the angiogenic factor most expressed in both TAT and SAT, followed by VEGF-B which was expressed by about 0.70 fold in TAT more than in SAT (Fig. 1A). However, VEGF-C and VEGF-D expression levels were very low compared to VEGF-A levels in both AT's. Western blotting analysis showed that in both TAT and SAT only VEGF-A (21kDa) and VEGF-B (64kDa) protein bands were detected (Fig. 1B), however no protein bands corresponding to VEGF-C and VEGF-D molecular weights were observed. Immunohistochemical analysis showed that in TAT, a considerable positive reaction for VEGF-A, VEGF-B and VEGF-C was detected; however in SAT we could only observe a positive reaction for VEGF-A and VEGF-B isoforms (Fig. 2). On the other hand, a strong positive correlation was found between VEGF-A and VEGF-C in TAT (***r*** = 0.803, p<0.005). In SAT, a positive correlation was detected between VEGF-A and VEGF-B (***r*** = 0.132, p<0.005), suggesting that TAT seems to have both angiogenic and lymphangiogenic properties.

### TAT Expressed High Levels of both COX-2 and HO-1 Compared to SAT

**Figure 3:**
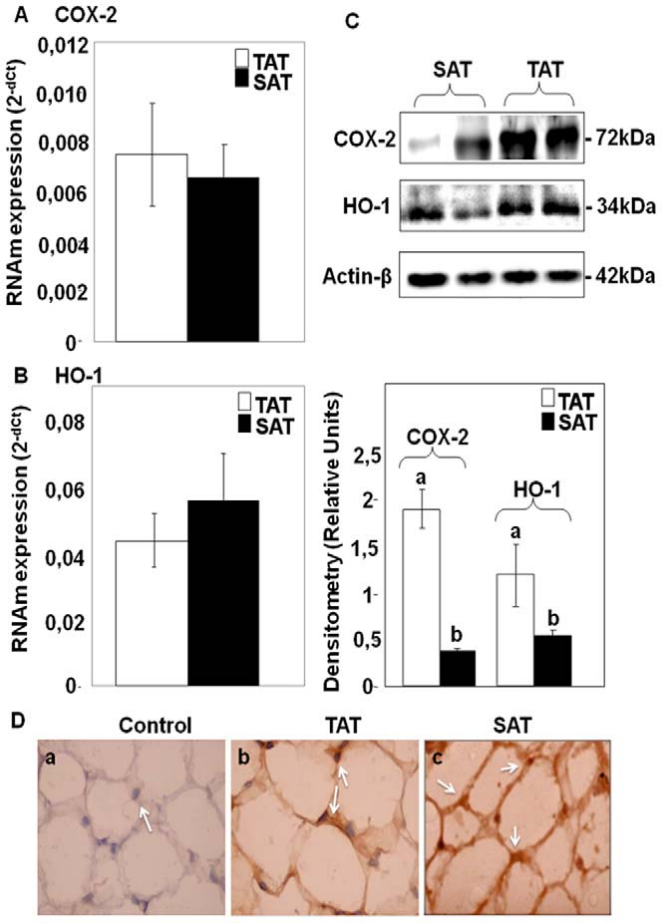
COX-2 and HO-1 mRNA and protein expressions in TAT and SAT. TaqMan® real time PCR for COX-2 and HO-1 mRNAs were performed on human adipose tissues (TAT and SAT). Specific mRNAs were normalized to cyclophilin (Cyc) levels. Results are expressed as the mean±SEM of experiment done in triplicate (n = 26) (A and B). Western blotting of COX-2 and HO-1 (C). Immunohistochemical identification of COX-2. Densitometry analyses are represented as a relative ratio of COX-2 and HO-1 to actin-β. Bars represent mean±SEM from 6 samples per adipose tissue type. Bars with different letters have a significant difference (P<0.05) (D).

In order to ascertain whether the expression of VEGF isoforms could be accompanied by inflammatory or anti-inflammatory mechanisms, we analyzed COX-2 and HO-1 levels in whole AT. Figure 3A shows that TAT significantly expressed high levels of COX-2 compared to SAT (p<0.05), while similar levels of HO-1 mRNA were detected in both AT's (Fig. 3B). Western blotting analyses show statistically significant (p<0.05) higher protein levels of both COX-2 and HO-1 in TAT compared to SAT where COX-2 and HO-1 protein levels were reduced by about 3.5 and 1.4 fold, respectively (Fig. 3C). Furthermore, immunostaining analyses show a positive reaction for COX-2 in the two AT's (Fig. 3D). Moreover, in TAT, a significant and strong correlation was found between VEGF isoforms and both COX-2 and HO-1 [***r***_(COX-2,VEGF-A)_ = 0.66, ***r***(_HO-1,VEGF-B)_ = 0.50, p<0.05] (Table 1), suggesting a potential connection between the mechanisms mediated by COX-2, HO-1 and VEGF-A/B expressions. However, no significant positive correlations were found between VEGF isoforms and COX-2 and HO-1 in SAT (Table 1).

**Table 1:** Correlation coefficients of VEGF isoforms withFABP-4, Adiponectin, PPARγ1/2, HIF-1α, COX-2 and HO-1.

Correlation coefficient, *r*								
N = 26	TAT				SAT			
Variables	VEGF isoforms				VEGF isoforms			
	A	B	C	D	A	B	C	D
**FABP4**	0.49^*^	0.81^*^	0.23	0.42	0.84	−0.10	−0.42	0.93
**ADIP**	0.69^*^	0.58^*^	0,18	0.24	0.40	−0.53	0.28	−0.04
**PPARγ1**	0.51	0.33	−0.04	0.03	0.68^*^	0.36	0.39	0.66
**PPARγ2**	0.63*	0.54*	0.12	0.31	0.72^*^	0.25^*^	0.46^*^	0.71
**HIF-1α**	0.38^*^	0.20^*^	−0.02	−0.71	−0.14	−0.22	0.41	0.32
**COX-2**	0.66^*^	0.19^*^	0.98^*^	0.07	0.46	0.54	−0.26	0.32
**HO-1**	0.20^*^	0.50^*^	0.66^*^	0.70	−0.64	0.34	0.57	−0.15

Correlation of VEGF isoforms mRNA expression with FABP-4, adiponectin (Adip), PPARγ1/2, COX-2 and HO-1 was determined by Spearman's correlation coefficient test (r). Strong correlation was demonstrated between VEGF isoforms and FABP-4, Adiponectin, PPARγ2, COX-2 and HIF-1α in thymus adipose tissue (TAT). However, with subcutaneous (SAT) adipose tissue a strong correlation was only detected between VEGF isoforms and PPARγ1/2. *p<0.05.

### Inflammatory and Anti-Inflammatory Marker Levels and the Degree of Macrophage Infiltration in TAT and SAT

**Figure 4:**
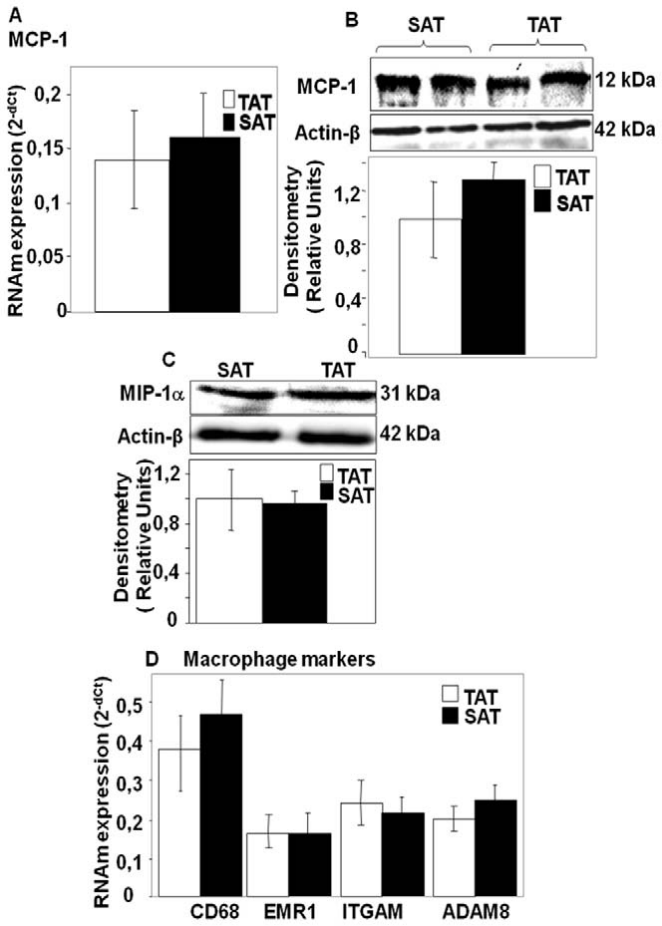
MCP-1, MIP-1α, ITGAM, EMR-1, CD68 and ADAM-8 mRNA and protein expression in TAT and SAT. TaqMan® real time PCR for MCP-1, MIP-1α, ITGAM, EMR-1, CD68 and ADAM-8 mRNAs was performed on human adipose tissues (TAT and SAT). Specific mRNAs were normalized to cyclophilin (Cyc) levels. Results are expressed as the mean±SEM of experiment carried out in duplicate (n = 26) (A,D). Western blotting of MCP-1 and MIP-1α. Densitometric analyses are represented as a relative ratio of MCP-1 and MIP-1α to actin-β. Bars represent mean±SEM from 6 samples per adipose tissue type(B and C).

In order to determine whether the high levels of COX-2 observed in TAT are the result of an elevated resident macrophage number in this AT, we analyzed the expression of classic inflammation markers, including monocyte chemoattractant protein-1 (MCP-1), a recruiting factor for circulating monocytes, chemokine macrophage inflammatory protein-1 alpha (MIP-1α), and macrophage markers CD68, CD11b, ITGAM, EMR1 and ADAM8. Figure 4 (A–D) shows that both SAT and TAT have similar mRNA and protein levels of MCP-1 and MIP-1. Furthermore, real time PCR and immunohistochemical analyses showed that all macrophage markers were also expressed equally in the two AT's (Fig. 4E), suggesting that the high levels of COX-2 observed in TAT are probably not due to the infiltration of a high number of resident macrophages in this AT, since the markers of this last fact were similar in both AT's.

On the other hand, and considering the cellular heterogeneity of TAT which would be responsible for the differentially expressed genes, we performed a flow cytometric analysis in order to determine the percentage of each cellular population. We observed that TAT contains a high proportion of cells that express FABP-4, which is a good marker of mature adipocytes [10] (Table 2). Macrophage cells, which showed a positive staining for CD11b, ITGAM and EMR1 represent about 0.25% of total thymus fat cells, endothelial cells about 15% and a very low percentage of thymocyte cells (0,05%). Next we analyzed the expression of genes known as mediators of adipogenesis, including PPARγ1, PPARγ2, FABP-4 and adiponectin in both TAT and SAT.

**Table 2:** Percentage of cell staining for cellular surface markers from adult thymus fat based on flow cytometric analysis.

Cell surface marker	% of positive cells
**FABP-4 (Fatty acid binding proteins)**	64.3±7,231
**CD11b, ITGAM, EMR1 (Macrophage markers)**	0.25±0.002
**CD45, CD31 (endothelial cell markers)**	15.8±2.010
**CD49, CD8, CD4 ( thymocyte markers)**	0.05±0.001

The data represents the mean of three experiments±SD. Each experiment consisted of a pool of cells from adult human thymus fat.

### PPARγ1, PPARγ2, FABP-4 and Adiponectin in Both SAT and TAT

**Figure 5:**
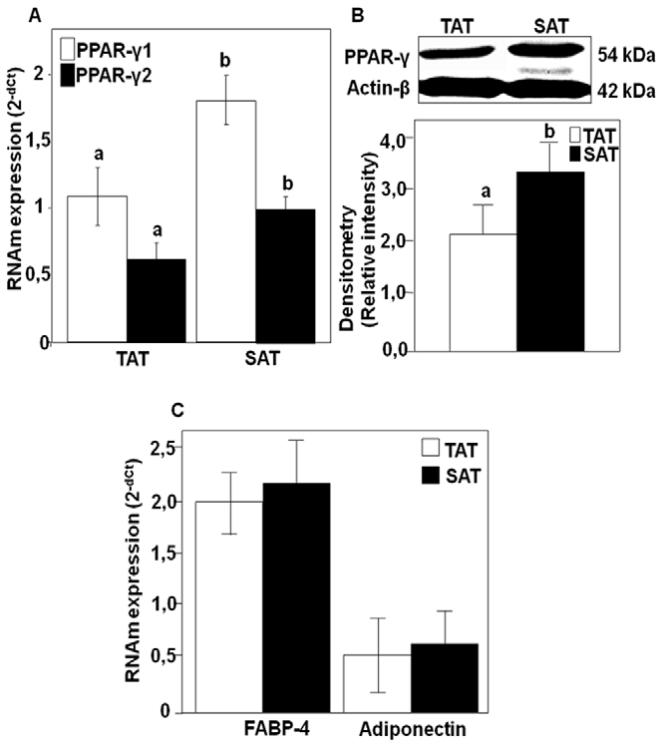
PPAR-γ FABP-4 and adiponectin mRNA and protein expression in TAT and SAT. TaqMan® real time PCR for PPAR-γ isoforms (PPAR-γ1 & PPAR-γ2) and FABP-4 and adiponectin was performed on TAT and SAT human tissues. PPARγ isoforms mRNAs were normalized to cyclophilin (Cyc) levels. Results are expressed as the mean±SEM of experiment done in duplicate (n = 26) (A and C). Immunoblotting analysis of PPARγ. Densitometry analyses are represented as a relative ratio of VEGF isoforms to actin-β. Bars represent mean±SEM from 6 samples per adipose tissue type. Bars with different letters have a significant difference, P<0.05(B).

Fig. 5A shows for the first time that TAT expresses both PPARγ isoforms (PPARγ1 and PPARγ2) with considerable levels compared to those observed in SAT. Also evaluating the PPARγ1/PPARγ2 ratio, we observed similar values (PPARγ2/γ1 = 0.518±0.02) in both AT's. On the other hand, strong correlations between VEGF-A and VEGF-B mRNA expression and PPARγ isoforms were only observed in SAT, [***r***_(PPARγ1,VEGF-A)_ = 0.68, ***r***_(PPARγ1,VEGF-B)_ = 0.36, ***r***_(PPARγ2,VEGF-A)_ = 0.72; ***r***_(PPARγ2,VEGF-B)_ = 0.25, p<0.05] (Table 1). By performing Western blot analysis on the two adipose tissues using antibodies specific for PPARγ, we could confirm the results obtained by quantitative mRNA expression analysis (Fig. 5B). Furthermore, expression analysis of adiponectin and FABP-4, genes known to regulate the adipogenesis process, demonstrated that both showed similar expression levels in both SAT and TAT (Fig 5C).

### HIF-1α mRNA and Protein Expression and DNA-Binding Activities of HIF-1α in TAT and SAT

**Figure 6:**
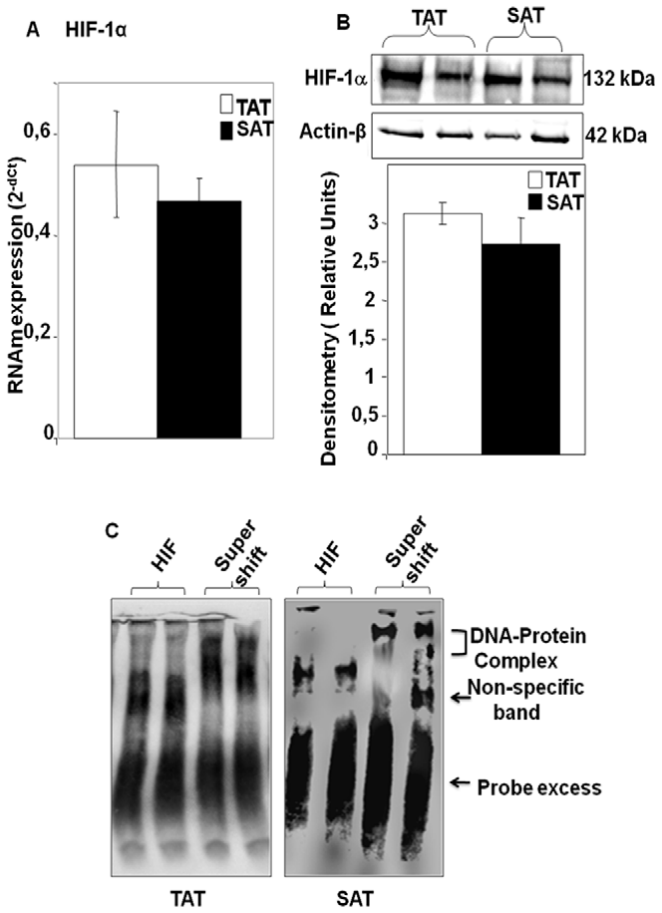
Hypoxia induced Factor -1α (HIF-1α) mRNA and protein expression and HIF-1 DNA-binding activity in TAT and SAT. TaqMan® real time PCR for HIF-1α mRNAs were performed on human adipose tissues (TAT and SAT). Specific mRNAs were normalized to cyclophilin (Cyc) levels. Results are expressed as the mean±SEM of experiments in duplicate (n = 26) (A). Western blotting of HIF-1α. Densitometric analyses are represented as a relative ratio of HIF-1α to actin-β. Bars represent mean±SEM from 6 samples per adipose tissue type (B). EMSA was performed by incubating AT nuclear extracts with a biotin labeled consensus HIF-1 probe from the human VEGF promoter region. Super shift was performed by the prior incubation of adipose tissue nuclear extract with anti-HIF-1α (super shift) and then EMSA was carried out by incubating with biotin labeled consensus HIF-1 (C). The blot is representative of three independent experiments with 8 samples from each adipose tissue type.

Hypoxia is known to induce angiogenesis, linking vascular oxygen supply to metabolic demand [11]. In recent years, accumulating evidence highlights the notion that hypoxia may exist in fat deposits as the tissue mass increases [12]. Here, mRNA expression analysis demonstrates that HIF-1α gene expression was induced equally in the two adipose tissues (Fig. 6A). Also, western blot analyses shows high levels of HIF-1α indicating the stability of this protein in both AT's (Fig. 6B), which signifies that both TAT and SAT seem to have similar hypoxic characteristics. On the other hand, a positive and significant correlation between VEGF isoforms and HIF-1α was observed in TAT (***r*** = 0.38, p<0.05), while no significant positive correlation between VEGF-C and HIF-1α was found in SAT (Table 1), suggesting the potential relationship between HIF-1 and VEGF expression regulation. For this reason we subsequently examined the binding activity of HIF-1 to the putative HRE-binding consensus in the promoter region of human VEGF gene by EMSA. As shown in figure 6C, nuclear extracts from TAT showed higher binding activity of HIF-1α to HRE consensus from the VEGF human gene promoter in TAT compared to SAT where the intensity of the shift bands was minor. The addition of anti-HIF-1α mAb resulted in an obvious super shift indicative of the union of HIF-1α to the DNA consensus region, suggesting the high DNA binding activity of HIF-1α transcription factor in TAT. As controls, nuclear extracts were incubated with a 100-fold excess unlabeled primer which resulted in complete loss of signal (data not shown).

## Discussion

Most of the studies on thymus fat are focused on immune senescence and various associated diseases related to atrophy and involution of this tissue [13]. Adult thymus specimens were obtained from cardiomyopathy ischemic patients undergoing cardiac surgery, due to the fact that part of this tissue is discarded in aortic cannulation procedures of the ascending aorta in cardiovascular surgery for patients in need of a cardiopulmonary bypass (CPB). However, until now, no studies have been carried out that consider thymus fat as a potential source of humoral and angiogenic factors, that could help in heart neovascularization after a cardiopulmonary bypass (CPB). Our idea is that thymus fat tissue and derived cells could serve as an angiogenic source which could help in the regeneration of new vessels in the ischemic myocardium. This hypothesis should be rigorously analyzed by studying the expression of the different angiogenic factors and especially those which are known to play relevant roles in angiogenesis and the generation of new vessels, and the molecular mechanisms that could have a crucial role in angiogenesis regulation in TAT. Here we present a preliminary and observational study which reveals the activation of a variety of genes and proteins generally known to be involved in inflammatory and anti-inflammatory processes and also in the regulation of angiogenic factors, especially the VEGF. Recently, we showed that thymus AT contains significant levels of a variety of angiogenic factors. Furthermore, developing cell migration and proliferation assays in which umbilical cord endothelial cells were evaluated for their ability to migrate and to proliferate, we observed an increase in proliferative and migratory activities in the presence of thymus extract [9], which are two relevant steps in angiogenesis, suggesting the angiogenic capacity of TAT. In the present work we observed that TAT expresses high levels of adipogenesis mediated genes, suggesting the generation of new adipose tissue in the thymus gland at old age. The expression of these adipogenic genes and those regulators of inflammation correlate positively with VEGF, suggesting the possible implication of these genes in VEGF regulation.

It is well known that over-expression of VEGF- A and VEGF- B has been associated with increased angiogenesis [14], while VEGF-C and VEGF-D are lymphangiogenic factors [15]. Here, evaluating the expression of VEGF isoforms, we found that TAT seems to have both angiogenic and lymphangiogenic properties, while SAT seems to be especially an angiogenic source, because VEGF-C expression was mostly detected in TAT. This VEGF-C positive staining suggests that the expression of this VEGF isoform could be related to the stromal lymphopoietin activity of Hassall's corpuscles that could conserve thymus AT [13]. In fact, here we could clearly appreciate the presence of these corpuscles after immunohistochemistry staining (data not shown), but we also observed that TAT showed a structure characterized by unilocular adipocytic cells such as subcutaneous adipose tissue. The next proposal of our study was to analyze whether VEGF expression could be accompanied by COX-2, a protein relevant in regulating pro-inflammatory processes, and HO-1, one of the most potent cytoprotector proteins, which have been demonstrated to play a relevant role in VEGF regulation [16]. In fact, works have shown that COX-2 promotes angiogenesis [17], and up-regulates VEGF expression [18]. Others have shown that HO-1 mediates VEGF stimulated angiogenesis [19]. Here we found that in the two AT's, a significant expression of COX-2 and HO-1 was detected, especially in TAT, suggesting that TAT displays the activation of both inflammatory and cytoprotective processes. Also the strong positive correlation of COX-2 and HO-1 with VEGF expression could suggest the potential relationship between these three proteins in TAT. These data have to be confirmed in future experiments, where signaling pathways should be well defined.

If we want to demonstrate that TAT is a white adipose tissue we have to define the different cell populations that constitute this tissue in order to determine whether we are studying a tissue that is 100% adipose or if it conserves its thymopoietic characteristics. For this end, we performed a cytometric analysis to characterize the different cell populations that constitutes TAT. As expected, TAT has a similar cellular profile as another white adipose tissue; in fact it contains a large amount of pre-adipocytes and adipocytes, a percentage of 15% of endothelial cells and 0.25% of macrophages. However and as expected, we could barely observe thymocytes either by cytometric analysis or by immunohistochemistry. Also, immunohistochemistry analysis showed a similar cellular structure as that observed in any other white adipose tissue. These data suggest that instead of talking about the degeneration of the thymic gland we should talk about the generation of new adipose tissue which replaces the generation of thymocytes. Also, the profile of gene expressions observed above could be the results of the presence of a variety of cell type in TAT. We believe that in the near future new experiments should be done in order to ascertain whether VEGF, COX-2 and HO-1 expressions are exclusive to adipocytes or other cells as macrophages and endothelial cells are involved in this genetic profile observed in whole TAT.

On the other hand, here we show that thymus tissues degeneration at old age is rather a substitution of thymocytes by adipocytes, indicating the presence of neo-generation mechanisms of new adipose tissue which is adipose tissue. In fact the analysis of the expression of a variety of genes known to be relevant mediators of adipogenesis and lipogenesis (PPARγ, adiponectin and FABP-4)(10,20–22), showed that both PPARγ1 and γ2 are expressed in both AT's. PPARγ2 is especially expressed in fat while PPARγ1 is expressed in fat and other tissues [20]. Recent works have demonstrated that PPARγ2 has a high adipogenic activity in adipose tissue [21], [22]. Also, adiponectin and FABP-4, two relevant adipogenesis mediators [10], [23], showed similar expression levels in TAT compared to those observed in SAT. Taken together, all these data show for the first time that TAT has adipocytic properties like other AT's. Furthermore, positive correlations were observed between PPARγ2, adiponectin, and FABP-4 expressions and VEGF-A and VEGF-B gene expressions, suggesting that the expression of these angiogenesis-related genes observed in this study could be largely localized in adipocytes than in other cell types such as macrophage or endothelial cells. Future experiments should be carried out to confirm this hypothesis, by separating the different cell types that constitute TAT and analyzing the expression of these genes in each one. Here we anticipate that both macrophages and adipocytes seem to have a potential role in the observed VEGF expressions, because strong positive correlations are also found between adipogenesis markers (PPARg2, adiponectin and FABP-4) and VEGF gene expression [r(PPARg2,VEGF-B) = 0.78*, r(FABP-4,VEGF-B) = 0.45**, r(adiponectin,VEGF-A) = 0.61**, **p<0.01 and *p<0.05)]. And also positive correlations are found between macrophage markers (MCP-1, ITGAM and EMR1) and VEGF gene expressions [r(MCP-1,VEGF-B) = 0.38**, r(ITGAM,VEGF-A) = 0.53*, r(EMR-1,VEGF-A) = 0.49*, **p<0.01 and *p<0.05)].

Also, in this study we analyzed another factor that characterizes adipose tissue, hypoxia induced factor-1(HIF-1). It is well known that adipose tissues show low oxygen pressures [12]. Furthermore, HIF-1 is a transcription factor that has been shown to be essential for VEGF transcriptional activation [24]. Here we found that HIF-1 from TAT recruits highly on the human VEGF promoter than from SAT, suggesting the potential implication of this transcription factor in the regulation of VEGF, especially in TAT. These results were confirmed by the detection of high levels of HIF-1α protein and a strong positive correlation between VEGF and HIF-1α in TAT. On the other hand, we found a significant positive correlation between HIF-1α and COX-2 [***r***_(COX-2, HIF-1)_ = 0.54, p<0.05) in TAT, suggesting the potential relationship between these two proteins in TAT. In conclusion, the fact that VEGF showed a positive correlation between both COX-2 and HIF-1 could lead us to believe that these two proteins may have a potential role in regulating VEGF, since in some white adipose tissues many authors have explained that VEGF activation implicates COX-2 gene expression activation through HIF-1α [25], because it has been observed that the COX-2 gene is directly transcriptionally activated in response to hypoxia by HIF-1 binding to a functional HRE within the proximal COX-2 promoter [26], and that COX-2 over-expression stimulates the expression of HIF-1, which in turn induces continued COX-2 expression [27].

Here we can conclude that adult thymus fat is an interesting source of angiogenic factors, which was previously unappreciated. Most studies have shown that the thymus loses its function during old age. With the data presented in this work we should say that the thymus replaces its immune function by another which is that of an ordinary white adipose tissue.

Moreover, it is evident that thymus fat presents a similar cellular profile as that observed in other white adipose tissues such as SAT. This cellular variety seems to be responsible for the observed differences in the expression of a variety of genes that are known to be relevant in the regulation of angiogenesis and adipogenesis in other white adipose tissues.

VEGF gene expression seems to be related to both adipocytes and macrophages, since the markers of these two cell types show a significant and strong correlation with VEGF gene expression in TAT. Also, this VEGF expression appears to be also associated with COX-2 and HIF-1 expression and activation in TAT. Our future objective is to well define the molecular mechanisms mediated by these proteins that could play a crucial role in VEGF regulation, in whole thymus adipose tissue and separately in each cell population.

One may wonder why use this thymus fat as a source of angiogenesis having access to other body fats such as subcutaneous and visceral adipose tissues. Firstly, as we mentioned previously, part of this fat is discarded during CBP surgery, and the results obtained in this and previous works [9] indicate that this tissue deserves to be studied in depth in order to investigate its potential usefulness as an adipose tissue. Furthermore, there are no adverse effects of removing this fat, because in our clinical experience these procedures had no surgical complications or morbidity associated. On the other hand, the largest source of thymus adipose tissue are ischemic patients, because part of this tissue used to be discarded in aortic cannulation procedures of the ascending aorta in cardiovascular surgery in patients in need of a cardiopulmonary bypass (CPB), and this is a procedure frequently used by cardiac surgeons [28], [29]. Furthermore, the studies done on thymus fat and thymus function are based in the vast majority in excising and removing thymus fat during cardiac interventions, which shows how often this practice is carried out, and its safety. Moreover, we are of the opinion that this fat could, in the future, provide much needed help in heart neovascularization after a cardiopulmonary bypass interventions (CPB), especially for patients who cannot benefit from coronary revascularization treatment after surgery or angioplasty [30].

## Supporting Information

Table S1 Clinicopathological characteristics and biomarker parameters in Ischemic Cardiomyopathy patients. The number of patients studied was 28. The mean age was 70.2±2.7 years. All patients received a coronary-artery bypass graft (CABG) with cardiopulmonary bypass (CPB). The mean number of grafts used was 3.1 per patient Values are presented as means±SE.
(0.03 MB DOC)
