# Supplementary material for: VEGF Gene Expression in Adult Human Thymus Fat: A Correlative Study with Hypoxic Induced Factor and Cyclooxigenase-2
Source: PLoS One. 2009 Dec 14;4(12):e8213. doi: 10.1371/journal.pone.0008213 (PMC2788242; doi:10.1371/journal.pone.0008213)
Supplement: Table S1 — Clinicopathological characteristics and biomarker parameters in Ischemic Cardiomyopathy patients. The number of patients studied was 28. The mean age was 70.2±2.7 years. All patients received a coronary-artery bypass graft (CABG) with cardiopulmonary bypass (CPB). The mean number of grafts used was 3.1 per patient Values are presented as means±SE. (0.03 MB DOC) [file pone.0008213.s001.doc]

**Table.S1.**

| **PARAMETERS** | **M/F (19/7)** |
| --- | --- |
| **Age (years)** | 70.20 ± 2.71 |
| **Glucose (mg/dL)** | 131.17 ± 12.02 |
| **Creatinine (mg/dL)** | 0.97 ± 0.06 |
| **Uric acid (mg /dL)** | 5.56 ± 0.47 |
| **Total cholesterol (mg/dL)** | 130.22 ± 10.48 |
| **HDL cholesterol (mg/dL)** | 28.78 ± 1.96 |
| **LDL cholesterol (mg/dL)** | 80.05 ± 8.22 |
| **Triglycerides (mg/dL)** | 146.00 ± 18.34 |
| **GOT (U/L)** | 26.39 ± 6.09 |
| **GPT (U/L)** | 50.83 ± 5.44 |
| **GGT (U/L)** | 51.33 ± 10.41 |
| **CRP (mg / L)** | 16.18 ± 4.54 |
| **Glycosylated Hb (%)** | 6.73 ± 0.40 |
